# Supplementary material for: Fridamycin A, a Microbial Natural Product, Stimulates Glucose Uptake without Inducing Adipogenesis
Source: Nutrients. 2019 Apr 1;11(4):765. doi: 10.3390/nu11040765 (PMC6520714; doi:10.3390/nu11040765)
Supplement: Supplementary file 1 [file nutrients-11-00765-s001.pdf]

Supplementary Materials

Fridamycin A, a Microbial Natural Product, Stimulates Glucose Uptake without Inducing Adipogenesis

Sun-Young Yoon [1,†], Seoung Rak Lee [1,†], Ji Young Hwang [1], René Benndorf [2], Christine Beemelmanns [2], Sang J. Chung [1,*] and Ki Hyun Kim [1,*]

[1] *School of Pharmacy, Sungkyunkwan University, Suwon 16419, Republic of Korea*

[2] *Leibniz Institute for Natural Product Research and Infection Biology – Hans-Knöll-Institute, Beutenbergstraße 11a, 07745 Jena, Germany*

Supporting Information Contents:

Figure S1. ESI-MS data of fridamycin A ……………………………………………………………………………S3
Figure S2. ^{1}H NMR spectrum of fridamycin A (CD_3OD, 800 MHz)……………………………………………S4
Experimental details……………………………………………………………………………………………S5-6

Figure S1. ESI-MS data of fridamycin A

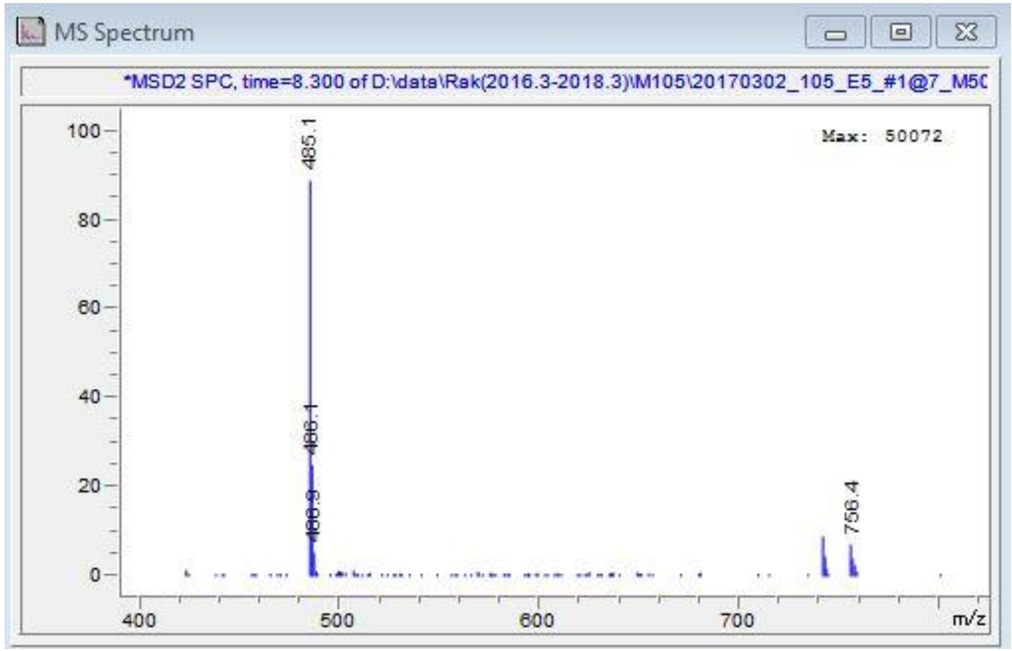

Figure S2. ^{1}H NMR spectrum of fridamycin A (CD_3OD, 800 MHz)

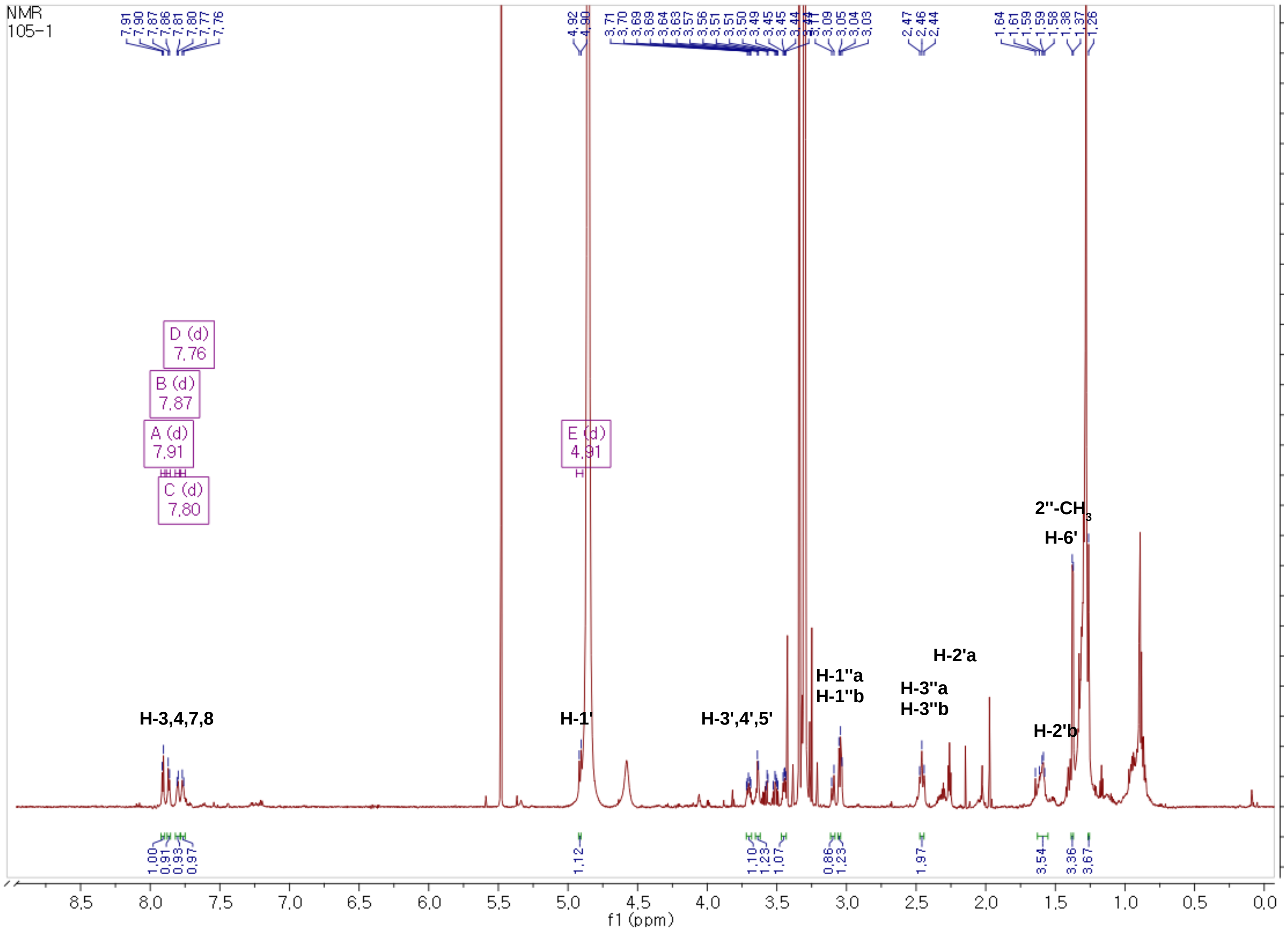

Experimental details

1. Microbial material

Actinomadura sp. RB99 was isolated from the surface of termite workers of the genus *M. natalensis* (colony Mn103, GPS S25 43 45.9 E28 14 08.9) in January 2010. Biomaterial was placed into clean plastic bags and processed within one day of collection. Termites were washed in sterile deionized water, and bacteria were isolated by plating the resulting suspensions on low-nutrient media with chitin (per liter: 4g chitin, 0.7 g K_2HPO_4, 0.3 g KH_2PO_4, 0.5 g $MgSO_4{\cdot}5H_2O$, 0.01 g $FeSO_4{\cdot}7H_2O$, 0.001 g $ZnSO_4$, 0.001 g $MnCl_2$, and 20 g agar) [1]. Isolates with *Actinobacteria*-like morphology were transferred to ISP2 agar (per liter: 10 g malt extract, 4 g yeast extract, 4 g glucose, and 20 g agar) and sub-cultured until pure isolates were obtained.

2. DNA extraction and PCR amplification

Actinomadura sp. RB99 was grown in nutrient-rich liquid ISP2 media for five to seven days at 30 °C. Cells were harvested, and genomic DNA was extracted using the GenJet genomic DNA purification kit (Thermo Scientific, #K0721) following the manufacturer's instructions with the following changes: (a) lysozyme treatment was extended to 40 min, and (b) proteinase K treatment was extended to 40 min. DNA was quantified photometrically using a Nanodrop Lite spectrometer (Thermo Scientific). For phylogenetic studies, the 16S rRNA gene was amplified using the primer set 1492R/27F. Each amplification reaction was prepared in a 25 µL final reaction volume containing 7.25 µL of distilled water, 5 µL of HF buffer, 5 µL of each primer (2.5 µM), 0.5 µL of dNTPs (10 µM), 0.25 µL of Phusion High-Fidelity DNA

polymerase (New England Biolabs), and 2 μL of extracted DNA (template). PCR was performed under the following conditions: 98 °C/38 s; 32 cycles of 98 °C/30 s, 52 °C/45 s, 72 °C/1 min 20 s; and a final extension at 72 °C/8 min. PCR product was visualized by agarose gel electrophoresis. PCR reaction products were purified using a PCR purification kit (Thermo Scientific). DNA fragments were sequenced at GATC (Konstanz).

3. Sequencing and species identification

Sequences were assessed for purity and mismatches using BioEdit [2]. The forward and reverse sequences obtained for each strain were assembled with BioEdit and tested for chimeras using DECIPHER (http://decipher.cee.wisc.edu/FindChimerasOutputs.html). Resulting sequences were deposited in GenBank (accession number: KY558684). Blast analyses with almost-complete 16S rRNA sequences (1368 bp) were performed using the NCBI database (reference RNA sequences). The results indicated that strain RB99 is a member of the genus *Actinomadura*. Sequences of the first 10 hits were downloaded from the NCBI database and aligned with the 16S rRNA sequence of *Actinomadura* sp. RB99 using the Sina sequences alignment service [3]. Two different phylogenetic trees were reconstructed with neighbor-joining or maximum-likelihood algorithms using MEGA software version 7.0.26 [4-6]. The evolutionary distance model of Tamura and Nei was used to generate evolutionary distance matrices for the maximum-likelihood and neighbor-joining algorithms, with the deletion of complete gaps and missing data [7]. For the maximum-likelihood algorithm, discrete gamma distribution was used (+*G*), and the rate variation model allowed for some sites to be evolutionarily invariable (+*I*). For the neighbor-joining algorithm, the rate variation among sites

was modeled with a gamma distribution (+*G*). The confidence values of nodes were evaluated by bootstrap analyses based on 1000 resampling steps [8].

References

1. Visser, A.A.; Nobre, T.; Currie, C.R.; Aanen, D.K.; Poulsen, M. Exploring the potential for actinobacteria as defensive symbionts in fungus-growing termites. Microb. Ecol. 2012, 63, 975-985.

2. Hall, T.A. BioEdit: a user-friendly biological sequences alignment editor and analysis program for Windows 95/98/NT. Nucleic Acids Symposium Series 1999, 41, 95-98.

3. Pruesse, E.; Peplies, J.; Glöckner, F.O. SINA: Accurate high-throughput multiple sequence alignment of ribosomal RNA genes. Bioinformatics 2012, 28, 1823-1829.

4. Saitou, N.; Nei, M. The neighbor-joining method: a new method for reconstructing phylogenetic trees. Mol. Biol. Evol. 1987, 4, 406-425.

5. Felsenstein, J. Evolutionary trees from DNA sequences: A maximum likelihood approach. J. Mol. Evol. 1981, 17, 368-376.

6. Kumar, S.; Stecher, G.; Tamura, K. MEGA7: molecular evolutionary genetics analysis version 7.0 for bigger datasets. Mol. Biol. Evol. 2016, 33, 1870-1874.

7. Tamura, K.; Nei, M. Estimation of the number of nucleotide substitutions in the control region of mitochondrial DNA in humans and chimpanzees. Mol. Biol. Evol. 1993, 10, 512-526.

8. Felsenstein, J. Confidence limits on phylogenies: an approach using the bootstrap. Evolution 1985, 39, 783-791.
